# Trends in the selection of insecticide resistance in Anopheles gambiae s.l. mosquitoes in northwest Tanzania during a community randomized trial of longlasting insecticidal nets and indoor residual spraying

**DOI:** 10.1111/mve.12090

**Published:** 2014-12-24

**Authors:** J. MATOWO, J. KITAU, R. KAAYA, R. KAVISHE, A. WRIGHT, W. KISINZA, I. KLEINSCHMIDT, F. MOSHA, M. ROWLAND, N. PROTOPOPOFF

**Affiliations:** ^1^ Department of Medical Parasitology and EntomologyKilimanjaro Christian Medical University CollegeMoshiTanzania; ^2^ Pan‐African Malaria Vector Research Consortium (PAMVERC)MoshiTanzania; ^3^ Department of Disease ControlLondon School of Tropical Medicine and HygieneLondonU.K.; ^4^ Amani Medical Research Centre, National Institute for Medical ResearchMuhezaTanzania; ^5^ Department of Infectious Disease EpidemiologyLondon School of Hygiene and Tropical MedicineLondonU.K.

**Keywords:** Ace‐1, bendiocarb, bioassays, carbamate, kdr, mutation, organophosphate, synergists

## Abstract

Anopheles gambiae s.l. (Diptera: Culicidae) in Muleba, Tanzania has developed high levels of resistance to most insecticides currently advocated for malaria control. The kdr mutation has almost reached fixation in An. gambiae s.s. in Muleba. This change has the potential to jeopardize malaria control interventions carried out in the region. Trends in insecticide resistance were monitored in two intervention villages using World Health Organization (WHO) susceptibility test kits. Additional mechanisms contributing to observed phenotypic resistance were investigated using Centers for Disease Control (CDC) bottle bioassays with piperonylbutoxide (PBO) and S,S,S‐tributyl phosphorotrithioate (DEF) synergists. Resistance genotyping for kdr and Ace‐1 alleles was conducted using quantitative polymerase chain reaction (qPCR). In both study villages, high phenotypic resistance to several pyrethroids and DDT was observed, with mortality in the range of 12–23%. There was a sharp decrease in mortality in An. gambiae s.l. exposed to bendiocarb (carbamate) from 84% in November 2011 to 31% in December 2012 after two rounds of bendiocarb‐based indoor residual spraying (IRS). Anopheles gambiae s.l. remained susceptible to pirimiphos‐methyl (organophosphate). Bendiocarb‐based IRS did not lead to the reversion of pyrethroid resistance. There was no evidence for selection for Ace‐1 resistance alleles. The need to investigate the operational impact of the observed resistance selection on the effectiveness of longlasting insecticidal nets and IRS for malaria control is urgent.

## Introduction

Current strategies for malaria vector control rely heavily on chemical means for both the indoor residual spraying (IRS) of insecticide and the use of insecticide‐treated nets (ITNs) [World Health Organization (WHO), 2013a]. Household ownership of longlasting insecticidal nets (LLINs) has been scaled up over the last few years in many countries in sub‐Saharan Africa (Flaxman *et al*., 2010; WHO, 2013a). Indoor residual spraying has been deployed in several areas of sub‐Saharan Africa, including in settings of high and medium rates of malaria transmission (Kolaczinski *et al*., 2007; Kigozi *et al*., 2012; Kim *et al*., 2012; Fullman *et al*., 2013). Unfortunately, the extensive use of insecticides has led to the selection of insecticide resistance in malaria vectors.

A number of countries in Africa, particularly those south of the Sahara, have deployed LLINs and IRS in combination in an attempt to further reduce malaria transmission (Kleinschmidt *et al*., 2009; Hamel *et al*., 2011; Corbel *et al*., 2012; West *et al*., 2014). Indoor residual spraying with non‐pyrethroid insecticides may be implemented in areas in which LLINs are used as part of an insecticide resistance management strategy (WHO, 2014). The WHO has recently advised that IRS should use non‐pyrethroid insecticides if LLINs and IRS are to be deployed together in the same geographical location in order to reduce selection pressure for pyrethroid resistance (WHO, 2014).

In order to reduce the burden of malaria morbidity and mortality in Tanzania, the National Malaria Control Programme (NMCP) adopted the WHO‐recommended strategies for the distribution and consistent use of LLINs, and spraying of houses with a safe and efficacious insecticide (IRS). To achieve high LLIN coverage, a variety of programmes including the Tanzania National Voucher Scheme (Mushi *et al*., 2003), the Under‐Five Catch‐up Campaign (Bonner *et al*., 2011), and the Universal Coverage Campaign (UCC, 2011) were implemented to target different at‐risk groups, and ‘hang‐up’ campaigns to improve net use were initiated.

As a result, the percentage of households in Tanzania that owned at least one ITN increased from 23% in 2005 to 92% in 2012 and the use of ITNs by household members increased from 15% in 2005 to 68% in 2012 [Tanzania Demographic and Health Survey (TDHS), 2005; Tanzania HIV/AIDS and Malaria Indicator Survey (THMIS), 2012].

According to the World Malaria Report of 2013, the number of people protected by IRS in Tanzania exceeded six million (WHO, 2013a). Indoor residual spraying started in Tanzania in 2007, targeting the Lake Zone, which had the highest burden of malaria. Pyrethroid‐based IRS with lambdacyhalothrin was launched in 2007 in the Muleba and Karagwe Districts of Kagera Region, which had the highest prevalences of malaria. An overall prevalence of 42% was documented in Muleba District in 2007–2008 (THMIS, 2008). Lambdacyhalothrin‐based IRS was changed to bendiocarb‐based IRS in Muleba in response to evidence of high pyrethroid resistance in the area (Protopopoff *et al*., 2013).

By 2010 reports of insecticide resistance in malaria vectors in Tanzania were scant (Mnzava, 1991; Kulkarni *et al*., 2007; Matowo *et al*., 2010). Two types of pyrethroid resistance (target site and metabolic) were reported in an *Anopheles arabiensis* population in northeast Tanzania (Kulkarni *et al*., 2006; Matowo *et al*., 2010). More recently, reports of DDT (dichlorodiphenyltrichloroethane) and pyrethroid resistance were published from other parts of the country, although susceptibility to organophosphates and carbamates persisted (Kabula *et al*., 2012, 2013, 2014; Protopopoff *et al*., 2013; Nkya *et al*., 2014). Both target site and metabolic mechanisms have been documented with the occurrence of L1014S and L1014F *kdr* mutations (target site mechanism) and the over‐transcription of genes encoding detoxification enzymes (metabolic mechanism).

Despite several years of pyrethroid‐based IRS and the increased use of LLINs in Muleba, *Anopheles gambiae s.s*. remains predominant, with the *kdr* mutation almost reaching fixation (Protopopoff *et al*., 2013). However, the role of metabolic involvement in the high level of pyrethroid resistance observed in the area remains unknown. Therefore, the objective of this study was to identify mechanisms underlying observed high phenotypic resistance to pyrethroids and DDT in *An. gambiae s.l*. from Muleba District and to assess trends of insecticide resistance in response to the campaign for universal coverage with LLINs and the change in vector control strategy from pyrethroid‐based IRS to carbamate‐based spraying. This study was performed as part of a 2‐year randomized controlled trial that evaluated the combined use of IRS and LLINs vs. that of LLINs alone for malaria control.

## Materials and methods

### Study area

**Figure 1:**
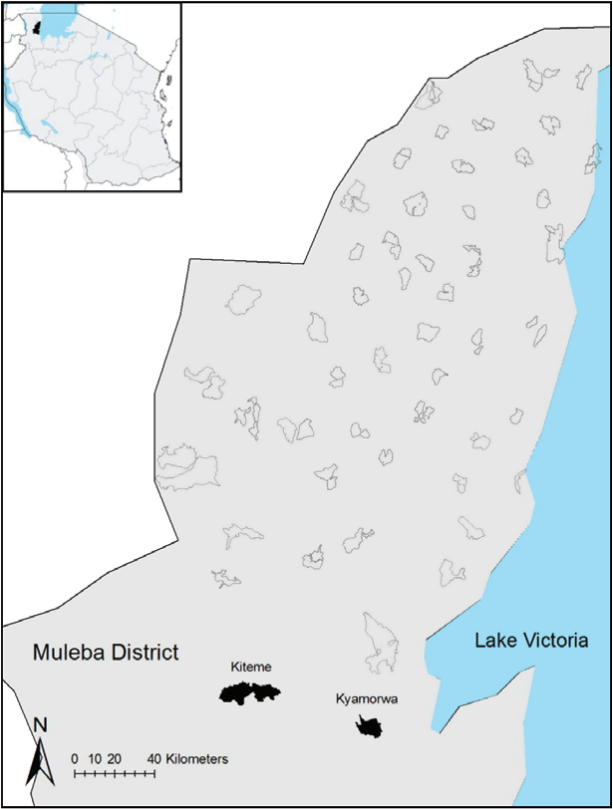
The map of Muleba District, in northwest Tanzania, showing the study sites at Kiteme and Kyamyorwa villages.

The study was conducted in two villages, Kyamyorwa (02°04′27.5″S, 31°34′10.8″E) and Kiteme (02°03′20.9″S, 31°27′16.8″E) in Muleba, a rural district on the western shore of Lake Victoria in northwest Tanzania (Fig. 1). Insecticide resistance monitoring was part of a large cluster randomized control trial evaluating the combined of IRS and LLINs against the use of LLINs alone (West *et al*., 2014). Both villages received the same interventions in 2011, which consisted of IRS with lambdacyhalothrin and universal coverage with LLINs. In 2012, Kiteme received LLINs only, whereas Kyamyorwa received LLINs and bendiocarb‐based IRS.

### Mosquito sampling

Resting adult mosquitoes were collected with aspirators early in the morning inside houses in Kyamyorwa and Kiteme villages in May and November/December of 2011 and 2012. Muleba has two rainy seasons interrupted by a distinct long dry season (June to August/September) and an unpredictable second dry season in December or January.

### WHO susceptibility tests

Batches of field‐collected, semi‐gravid *An. gambiae s.l*. mosquitoes were exposed to diagnostic dosages of the three pyrethroids (0.05% lambdacyhalothrin, 0.05% deltamethrin, 0.75% permethrin), one organochlorine (4% DDT), one carbamate (0.1% bendiocarb) and one organophosphate (0.1% pirimiphos‐methyl) using standard WHO susceptibility test kits for adult mosquitoes (WHO, 2013b).

Mosquitoes were exposed for 1 h and then transferred to holding tubes and provided with 10% glucose solution for 24 h, after which mortality was recorded. Tests in which control mortality exceeded 5% were excluded.

### Synergy studies

Synergy tests using Centers for Disease Control (CDC) bottle bioassays were conducted in unfed female mosquitoes representing the F1 progeny of wild‐caught *An. gambiae s.l*. and aged 3–5 days. The glass bottles were coated with permethrin or one or more synergists as described by Brogdon and McAllister (1998). Piperonylbutoxide (PBO) and S,S,S‐tributyl phosphorotrithioate (DEF) were used as synergists to inhibit mixed‐function oxidases and non‐specific esterases, respectively. Four treatments were compared during each test run: permethrin alone; permethrin plus DEF; permethrin plus PBO, and permethrin plus both PBO and DEF. The control bottle was treated with acetone alone.

The mosquitoes were pre‐exposed to synergists for 1 h before they were transferred into bottles treated with permethrin (21.5 μL/mL/bottle). Control mosquitoes were pre‐exposed in an untreated bottle for 1 h. Knock‐down was recorded at 5‐min intervals for 60 min. Mosquitoes were then transferred to holding cups, supplied with glucose solution and mortality recorded after 24 h. The same tests were conducted in an *An. gambiae s.s*. susceptible strain (Kisumu). Ten replicates of 10 mosquitoes were exposed to each treatment.

### Knock‐down resistance (kdr) and Ace‐1 genotyping

Genomic DNA was extracted using Chelex‐100 from the legs of both mosquitoes that survived and those that died following exposure to pyrethroids, DDT and bendiocarb. The DNA was stored at −20 °C until use. The hydrolysis probe/TaqMan assay (Bass *et al*., 2007) was used with minor modifications in the cycling conditions to detect *kdr* and *Ace‐1* mutations.

### Data analysis

Percentage mortalities at 24 h post‐exposure were used to assess the status of susceptibility/resistance to insecticides, according to the WHO criteria (WHO, 2013b). The 95% confidence intervals (CIs) of the percentage mortality were calculated using the binomial exact method as implemented in the *cci* command in stata Version 11.0 (Stata Corp. LP, College Station, TX, U.S.A.). The KdT_50_ and 95% CI values in synergy studies were calculated for each treatment using Probit analysis in pasw Statistics for Windows Version 18.0 (SPSS, Inc., Chicago, IL, U.S.A.).

### Ethical considerations

Ethical clearance was given by the ethics review committees of the Kilimanjaro Christian Medical College, the National Institute for Medical Research Tanzania and the London School of Hygiene and Tropical Medicine (Application no. 5814). Written informed consent was obtained from community leaders and the households from which the mosquitoes were collected.

## Results

### Insecticide susceptibility tests

**Figure 2:**
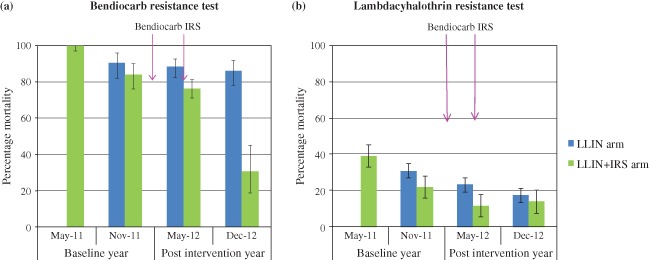
Trends in the resistance status of Anopheles gambiae s.l. populations in the two villages exposed to bendiocarb and lambdacyhalothrin in May and November in 2011 and 2012. No susceptibility tests were conducted in the village supplied with longlasting insecticidal nets (LLINs) in May 2011. IRS, indoor residual spraying.

In 2011 before the administration of bendiocarb‐based IRS, the percentage mortality after exposure to lambdacyhalothrin test papers was 31% in the LLIN village and 22% in the LLIN + IRS village. The percentage mortality decreased in the two villages to 17% and 14%, respectively, in December 2012 (Fig. 2). Resistance to other pyrethroids (permethrin and deltamethrin) was also observed, with test mortality always < 35% (Table 1).

**Table 1:** Mortality rates in Anopheles gambiae s.l. populations sampled from the two villages in the longlasting insecticidal nets (LLINs) only and the LLINs with indoor residual spraying (IRS) arms of the intervention in response to diagnostic concentrations of permethrin, deltamethrin, lambdacyhalothrin, DDT, bendiocarb and pirimiphos‐methyl in World Health Organization (WHO) susceptibility tests

	Number exposed (% mortality)					
	Kiteme (LLINs only)			Kyamyorwa (LLINs + IRS)		
Insecticide	November 2011	May 2012	December 2012	November 2011	May 2012	December 2012
DDT	85 (40%)	104 (18%)	—	99 (13%)	95 (13%)	—
Permethrin	98 (11%)	101 (22%)	—	—	119 (15%)	—
Deltamethrin	—	99 (22%)	—	—	82 (34%)	—
Lambdacyhalothrin	104 (31%)	159 (23%)	75 (17%)	232 (22%)	183 (12%)	29 (14%)
Bendiocarb	84 (91%)	161 (88%)	114 (86%)	175 (84%)	325 (76%)	52 (31%)
Pirimiphos methyl	—	10 (100%)	—	—	101 (100%)	—

Subsequent to tests of bendiocarb in the pre‐intervention year (2011), mortality rates rose to > 80% in both villages. A decrease in test mortality was observed in the LLIN + IRS village in the intervention year (2012), whereby test mortality decreased from 84% in November 2011 to 76% in May 2012 and 31% in December 2012 after the introduction of IRS with bendiocarb (Fig. 2). Test mortality with bendicarb papers in the LLIN village remained above 80% in December 2012. Tests with the organophosphate pirimiphos‐methyl showed 100% susceptibility in both villages in May 2012.

### Synergy studies

**Figure 3:**
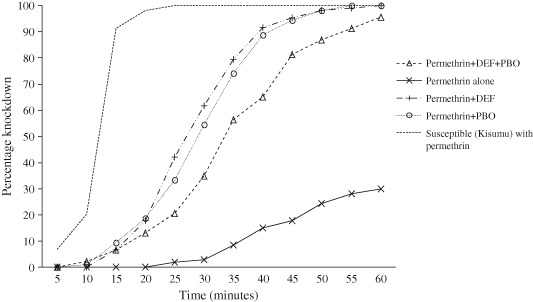
Percentage knock‐down over time for an F1 generation of wild‐caught mosquitoes exposed to permethrin alone, permethrin + piperonylbutoxide (PBO), permethrin + S,S,S‐tributyl phosphorotrithioate (DEF), and permethrin + PBO + DEF relative to a permethrin test of a susceptible mosquito strain (Kisumu) in Centers for Disease Control (CDC) bottle bioassays.

The knock‐down time at KT_50_ when exposed to permethrin alone was higher in the F1 *An. gambiae s.l*. adult females than in the susceptible strain (Kisumu), giving a resistance ratio of 5.4. Exposure of F1 *An. gambiae s.l*. adult females to permethrin plus PBO or DEF resulted in a higher percentage knock‐down at each time interval in comparison with exposure to permethrin alone (Fig. 3). Permethrin plus the PBO and DEF mixture achieved a consistently lower percentage knock‐down than permethrin plus PBO alone or DEF alone; synergy ratios ranged from 2.8 to 3.5.

The mortality rate after 24 h for F1 *An. gambiae s.l*. was 17.8% for permethrin alone, but ranged from 83.2% to 96.6% with the addition of PBO, DEF or both, and therefore confirmed the synergy (Table 2).

**Table 2:** Synergy tests for Anopheles gambiae s.l. (F1) from Muleba relative to the standard susceptible strain (Kisumu) in Centers for Disease Control (CDC) bottle bioassays

	Mortality, % (*n*)		KdT_50_, min (95% CI)		
Treatment	F1 wild progeny	Susceptible strain	F1 wild progeny	Susceptible strain	KdT_50_ ratio
Permethrin alone	17.8% (107)	100% (118)	62.9 (59.6–67.4)	11.6 (10.7–12.4)	5.4
Permethrin + DEF	83.2% (107)	100% (95)	28.1 (27.0–29.1)	8.5 (6.7–10.2)	3.3
Permethrin + PBO	93.5% (108)	100% (107)	28.9 (28.1–29.7)	10.3 (9.8–10.8)	2.8
Permethrin + PBO + DEF	96.6% (92)	100% (98)	34.8 (33.8–35.9)	10.0 (9.5–10.4)	3.5

PBO, piperonylbutoxide; DEF, S,S,S‐tributyl phosphorotrithioate.

### Knock‐down (kdr) and Ace‐1 genotyping

The frequency of the leucine–serine point mutation (*kdr‐e*) was the same in the two villages at baseline (high allelic frequency: 0.98) (Fisher's exact test, *P* = 0.596) and during the intervention year (Fisher's exact test, *P* = 0.635), presumably because the *kdr* mutation had almost reached fixation in the predominant *An. gambiae s.s*. population. No altered (insensitive) acetylcholinesterase was found in any of the mosquitoes screened for the *Ace‐1* (G119S) mutation.

## Discussion

The present study explored trends in the selection of insecticide resistance phenotypes in *An. gambiae s.l*. in northwest Tanzania during a two‐arm cluster randomized trial in which one study site received LLINs only and the other received both LLINs and bendiocarb‐based IRS (Protopopoff *et al*., 2013; West *et al*., 2014). Prior to the interventions, IRS with lambdacyhalothrin had been sprayed annually for several years, including the baseline year of 2011. High‐frequency phenotypic resistance to DDT and to each of the pyrethroids tested was already present in the baseline year, presumably as a result of recurrent IRS campaigns because nowhere else in Tanzania was such high resistance observed at that time (Kabula *et al*., 2013) and this was the only region to be put under intensive pyrethroid IRS.

In 2011, before the delivery of any bendiocarb‐based IRS, low‐level resistance to bendiocarb was known to exist in the area. In the village that was not sprayed in 2012, the bendiocarb test mortality rate remained constant between 2011 (91%) and 2012 (86%). However, in the village that was sprayed with the carbamate, there was a marked decrease in bendiocarb test mortality after the second round of spraying from 76% at baseline to 31% in December 2012 as a result of the selection of bendiocarb‐resistant vectors.

It is notable that differential selection of carbamate resistance between the sprayed and non‐sprayed neighbouring clusters was evident, indicating that selection for resistance in the IRS village was stronger than the rate of dispersal of bendiocarb‐resistant mosquitoes between clusters; thus the increase in the proportion of bendiocarb‐resistant mosquitoes can be attributed to the IRS. The cause of the pre‐existing resistance in 2011 is unknown. There is some possibility of the development of cross‐resistance between carbamates and pyrethroids, but this has yet to be studied.

In the village that received LLINs, the frequency of resistance to bendiocarb recorded before the intervention did not change in 2012, which indicates that no further selection pressure was exerted.

The practice of using insecticide rotations as a strategy to avoid the development of resistance requires the deployment of two or more insecticide classes over time to reduce selection pressure for resistance to any single class. The conditions required for rotations to delay selection for resistance are that resistance alleles are deleterious in the absence of insecticide selection and that residues of the selecting insecticide have decayed to a point at which they no longer select (Denholm & Rowland, 1992). In Muleba during the period in 2011 and 2012 when pyrethroid‐based IRS was ceased, there was no evidence for any decrease in the frequency of pyrethroid resistance when carbamate‐based IRS was introduced. However, the problem faced in Muleba concerns the continued presence of pyrethroid in the form of universal coverage of LLINs. No reversal can be expected in this context if the LLINs continue to select for pyrethroid resistance. There is evidence that LLINs did continue to select for pyrethroid resistance as the proportion of resistant survivors in pyrethroid susceptibility tests continued to increase in the LLIN village between November 2011 and December 2012. The trend in the LLIN + IRS village was not dissimilar. While LLINs are used, there is no possibility of reversing resistance to pyrethroids.

The existing data provide no evidence on whether the resistance is deleterious or neutral in the absence of insecticide selection because the conditions required to test this are unavailable as LLINs are still in use. It is also possible that pyrethroid resistance in the Muleba *An. gambiae s.l*. population was too high (Protopopoff *et al*., 2013), and that pyrethroid resistance will revert after many generations. However, reversal cannot be expected if the LLINs continue to select for pyrethroid resistance. Nevertheless, it is clear that bendiocarb‐based IRS succeeded in killing pyrethroid‐resistant mosquitoes and reducing longevity and hence facilitated a reduction in malaria transmission (West *et al*., 2014).

Bendiocarb is one of the few residual insecticides approved by the WHO which can serve as a potential alternative against pyrethroid‐resistant *An. gambiae*, and large‐scale IRS campaigns using bendiocarb are being scaled up in several West and East African countries through the President's Malaria Initiative (PMI, 2009; Akogbéto *et al*., 2010). Elsewhere in West Africa, new alternative insecticides such as chlorfenapyr are being tested for the control of pyrethroid‐resistant mosquitoes in experimental huts (N'Guessan *et al*., 2009; Ngufor *et al*., 2011). Experimental hut studies provide some empirical data in support of interventions which combine the use of LLINs and IRS and indicate that these constitute a potential tool for the management of vector resistance (Djènontin *et al*., 2010; Ngufor *et al*., 2011, 2014), but data on the differential survival of alleles or resistance and susceptibility in these small‐scale hut studies are too limited to be conclusive.

The present study found no further selection for the *kdr*‐based resistance to pyrethroids between the pre‐ and post‐intervention years and neither was the *Ace‐1* (G119S) allele, which confers resistance to organophosphates and carbamates, detected. There is a need to investigate the underlying mechanism that confers resistance to bendiocarb, its impact on malaria control in the area, and whether it is the same metabolic mechanism that confers additional resistance to pyrethroids. The reduced susceptibility to bendiocarb (carbamate) recorded before the implementation of bendiocarb‐based IRS in Muleba District (Protopopoff *et al*., 2013) cannot be linked to the agricultural use of insecticides because the localities in which the study was conducted are non‐agricultural.

The fact that no *Ace‐1* (G119S) mutation was observed in the Muleba *An. gambiae s.l*. populations suggests the potential involvement of metabolic resistance mechanisms and/or an alternative mutation associated with G119S. Elevated levels of esterases have been reported to represent a primary mechanism involved in organophosphate and carbamate resistance (Hemingway & Karunaratne, 1998; Hemingway *et al*., 2004) and were reported to play a role in the development of resistance to bendiocarb in *An. gambiae s.l*. populations in the south–north transect of Benin (Aïzoun *et al*., 2013). The carbamate resistance in Muleba could also be potentially conferred by P450s and there is a possibility of a cross‐resistance between pyrethroids and carbamate (Brooke *et al*., 2001; Edi *et al*., 2014). Resistance to permethrin was partially suppressed by PBO and DEF in the Muleba strain, confirming the involvement of P450s monooxygenases and esterases in resistance. Several studies have reported that both monooxygenase and esterase are involved in bendiocarb resistance (Cochran, 1987; Scott *et al*., 1990; Lee *et al*., 1996).

The high levels of these detoxification enzymes in Muleba may be associated with selection pressure that resulted from pyrethroid IRS that has been implemented in the area since 2007. The scaling‐up of permethrin Olyset LLINs under the Universal Coverage Campaign (UCC, 2011) is most likely increasing selection for higher levels of both esterases and oxidases and maintaining high resistance to pyrethroid in the LLINs village. A previous study in Kenya (Mathias *et al*., 2011) suggested that the agricultural use of insecticides may play a role in the evolution of bendiocarb resistance in malaria vectors in western Kenya, although ITNs have imposed the most important selection pressure in the area. Such selection pressure may have resulted in elevated levels of oxidases or both oxidases and esterases in western Kenyan *An. gambiae s.s*. populations (Kawada *et al*., 2011; Ochomo *et al*., 2013). Elsewhere in Uganda a few kilometres north of our study area, malaria vectors remain fully susceptible to bendiocarb (Morgan *et al*., 2010; Mawejje *et al*., 2013). However, biochemical assays and synergy studies suggest the involvement of P450 monooxygenases in pyrethroid resistance in these vectors. The absence of bendiocarb resistance in Uganda implies that elevated levels of esterases are more important than those of oxidases in conferring carbamate resistance, as previously documented by Hemingway and Karunaratne (1998) and Hemingway *et al*. (2004). The *An. gambiae s.l*. Seme populations in Benin remained susceptible to bendiocarb at 2 years after a fourth round of bendiocarb IRS (Padonou *et al*., 2012).

Selection for carbamate resistance as a result of the IRS intervention does not necessarily mean that bendiocarb‐based IRS will shortly become ineffective. During the course of the intervention year, bendiocarb‐based IRS proved highly effective and succeeded in reducing the overall prevalence of malaria by 57% (West *et al*., 2014). At the start of the intervention, 84% of *An. gambiae s.l*. were still susceptible to carbamates and during the course of the intervention, while resistance was being further selected, the carbamate‐based IRS still managed to control the transmission of malaria satisfactorily. The question of interest concerns whether the high frequency of resistance recorded at the end of the intervention (when only 31% of *An. gambiae s.l*. remained susceptible) would be sufficient to start impairing control should bendiocarb‐based IRS continue.

Impairment would depend on the strength of the resistance (the resistance ratio) rather than the frequency of resistance, and that strength has yet to be measured. Whether or not carbamate resistance marks the beginning of the end of bendiocarb use in northwest Tanzania, there appears to be no cross‐resistance between this mechanism and organophosphates, and longlasting organophosphates should prove effective if introduced (N'Guessan *et al*., 2010; Oxborough *et al*., 2014).

Currently, the IRS strategy in Tanzania is preferentially based on the use of carbamates, especially in areas in which pyrethroid resistance has been reported. The spread of resistance may represent a major threat to the effectiveness of this strategy. Complementary studies are needed to determine whether bendiocarb resistance has spread to other regions, especially those with high prevalences of malaria and those which are prone to malaria epidemics.

## Conclusions

The presence of multiple resistance mechanisms in *An. gambiae s.l*. was revealed in northwestern Tanzania. Combined metabolic and *kdr*‐based resistance mechanisms represent a serious threat to the effectiveness of current malaria vector control operations based on the use of LLINs and IRS. The continuing susceptibility of *An. gambiae s.l*. to organophosphates indicates that longlasting formulations of insecticides such as pirimiphos methyl may be suitable for IRS in combination with LLINs. There is an urgent need to investigate the operational impact of the multiple mechanisms of resistance on the efficacy of LLINs, the LLINs which incorporate PBO synergists, and the future use of actellic (organophosphate) IRS. The extent and diversity of insecticide resistance phenotypes and their underlying mechanisms in *An. gambiae s.l*. mosquitoes call for new alternative strategies to complement current insecticide‐based protocols and to reduce reliance on these alone.

## Authors' contributions

JM conceived the study design, performed the experiments, carried out data analysis and interpretation and wrote the first draft of the manuscript. JK participated in data analysis and interpretation. RK, RAK, AW were involved in quantitative real time PCR for *Ace‐1* genotyping. WK, IK and FM critically revised the manuscript and facilitated the implementation of the work. MR was involved in study design, interpretation of the data and revision of the manuscript. NP was involved in the study design, implementation and supervision of the data collection, data analysis and interpretation and revised the manuscript. All authors have read and approved the final version of the manuscript.
